# Association of *ALDH2* Genotypes and Alcohol Intake with Dietary Patterns: The Bunkyo Health Study

**DOI:** 10.3390/nu14224830

**Published:** 2022-11-15

**Authors:** Mari Sugimoto, Hiroki Tabata, Hideyoshi Kaga, Yuki Someya, Saori Kakehi, Abulaiti Abudurezake, Hitoshi Naito, Naoaki Ito, Huicong Shi, Hikaru Otsuka, Futaba Umemura, Yasuyo Yoshizawa, Ryuzo Kawamori, Hirotaka Watada, Yoshifumi Tamura

**Affiliations:** 1 Department of Sports Medicine and Sportology, Graduate School of Medicine, Juntendo University, 2-1-1 Hongo, Bunkyo-ku, Tokyo 113-8421, Japan; 2 Sportology Center, Graduate School of Medicine, Juntendo University, 2-1-1 Hongo, Bunkyo-ku, Tokyo 113-8421, Japan; 3 Metabolism and Endocrinology, Graduate School of Medicine, Juntendo University, 2-1-1 Hongo, Bunkyo-ku, Tokyo 113-8421, Japan; 4 Center for Healthy Life Expectancy, Graduate School of Medicine, Juntendo University, 2-1-1 Hongo, Bunkyo-ku, Tokyo 113-8421, Japan; 5 Faculty of International Liberal Arts, Juntendo University, 2-1-1 Hongo, Bunkyo-ku, Tokyo 113-8421, Japan

**Keywords:** dietary patterns, *ALDH2* genotype, alcohol intake

## Abstract

Dietary habits are associated with various diseases and assessed by dietary patterns (DPs). Since the *ALDH2* genotype is correlated with alcohol and several food preferences, this genotype is probably associated with DPs. In this cross-sectional study of 1612 elderly adults, we investigated the effects of the *ALDH2* genotype on DPs and the mediating role of alcohol intake. We identified the *ALDH2* genotype and conducted a dietary history survey, then used principal component analysis to determine DPs for each gender. We performed multiple regression analysis to determine the independent contribution of the *ALDH2* genotype and alcohol intake to DP scores. We identified three DPs: the “Japanese side dish type” (DP1), the “Japanese dish with alcohol type” (DP2), and the “Western dish with alcohol type” (DP3). In men, the single nucleotide polymorphism *ALDH2* rs671 was significantly associated with all DP scores. When alcohol intake was added as a covariate, *ALDH2* rs671 was still significantly correlated with the DP2 score but not with the DP1 or DP3 score, and alcohol intake was significantly correlated with all DP scores. In women, *ALDH2* rs671 was significantly associated with the DP2 and DP3 scores; however, after adding alcohol intake as a covariate, these associations disappeared, and alcohol intake significantly correlated with all DP scores. In conclusion, the *ALDH2* genotype was associated with several DPs in elderly adults, but most associations were mediated by alcohol intake.

## 1. Introduction

Dietary habits are associated with the development of various diseases and are assessed by dietary patterns (DPs) [1,2,3,4,5,6]. DPs are statistically evaluated by dietary survey data on the quantities, proportions, varieties, and combinations of different foods, drinks, and nutrients in diets, and the frequency with which they are habitually consumed [1,5,6]. Since people obtain nutrition from multiple foods, it would be beneficial to consider how the risk of disease development is affected by interactions between nutrients and their synergistic effects. In fact, DPs may be more predictive of disease risk than single nutrients or foods [2,3,4]. Additionally, in clinical intervention trials, changes in DPs appeared to be more effective than single-nutrient interventions [7,8]. Thus, clarifying DPs may facilitate the individualized risk prediction of disease development and help optimize dietary interventions.

Previous reports showed that the single nucleotide polymorphism aldehyde dehydrogenase 2 gene (*ALDH2*) rs671 is associated with alcohol intake [9,10,11]. Ethanol is oxidized by alcohol dehydrogenase to acetaldehyde, which is subsequently oxidized by ALDH2 to acetic acid. The *ALDH2* rs671 G allele encodes Glu at amino acid 504, resulting in the enzymatically active form, and the *ALDH2* rs671 A allele encodes Lys at amino acid 504, yielding the enzymatically inactive form [12]. Approximately 99% of Caucasians are *ALDH2* rs671 G homozygotes [13], compared to only about 55% of Japanese individuals. Individuals with *ALDH2* rs671 A/A metabolize toxic acetaldehyde more slowly, resulting in side effects such as flushing, nausea, and vomiting after alcohol intake; therefore, alcohol intake usually depends on the *ALDH2* genotype [9,10,11]. Volumes of alcohol intake in Japanese men who were *ALDH2* rs671 G/G, G/A, or A/A carriers were found to be ~28 g/day, ~13 g/day, and 1 g/day, respectively [10].

*ALDH2* rs671 is also associated with individual dietary habits [5,14,15,16,17,18,19]. The A allele of *ALDH2* rs671 is associated with increased coffee, tea, milk, yogurt, and sweet food intake, and with decreased fish, natto, tofu, and alcohol intake [9,14,15,18,19]. Given these numerous correlations between *ALDH2* rs671 and food and beverage intake, *ALDH2* rs671 may also be associated with DPs, although this remains unproven. On the other hand, several previous studies have reported that many DPs are characterized by alcohol intake [20,21,22,23,24,25,26]. Therefore, even if the *ALDH2* genotype is associated with DPs, alcohol intake may be an intermediate factor. Given that the *ALDH2* rs671 G allele is associated with increased blood glucose, blood pressure, and high-density lipoprotein cholesterol [27,28,29], clarifying the relationships between the *ALDH2* genotype, alcohol consumption, and DPs may be beneficial for preventing diseases related to these clinical parameters.

Against this background, the purpose of this study was to examine the association between DPs and *ALDH2* gene polymorphisms in community-dwelling Japanese elderly adults who participated in the Bunkyo Health Study [30]. We hypothesized that the *ALDH2* rs671 would be associated with DPs and that even if the *ALDH2* genotype was associated with DPs, this association would be intermediated by alcohol intake. 

## 2. Method

### 2.1. Study Design and Participants

This cross-sectional study used the baseline data of the Bunkyo Health Study [30]. Briefly, we recruited individuals aged 65–84 years living in Bunkyo-ku, an urban area in Tokyo, Japan, at the Sportology Center of Juntendo University from 15 October 2015 to 1 October 2018. Exclusion criteria consisted of pacemaker or defibrillator placement and diabetes requiring insulin therapy. After an overnight fast, participants underwent body composition measurement by bioelectrical impedance analysis (InBody770, InBody Japan, Tokyo, Japan) and fasting blood sampling, followed by a 75 g oral glucose tolerance test.

**Figure 1:**
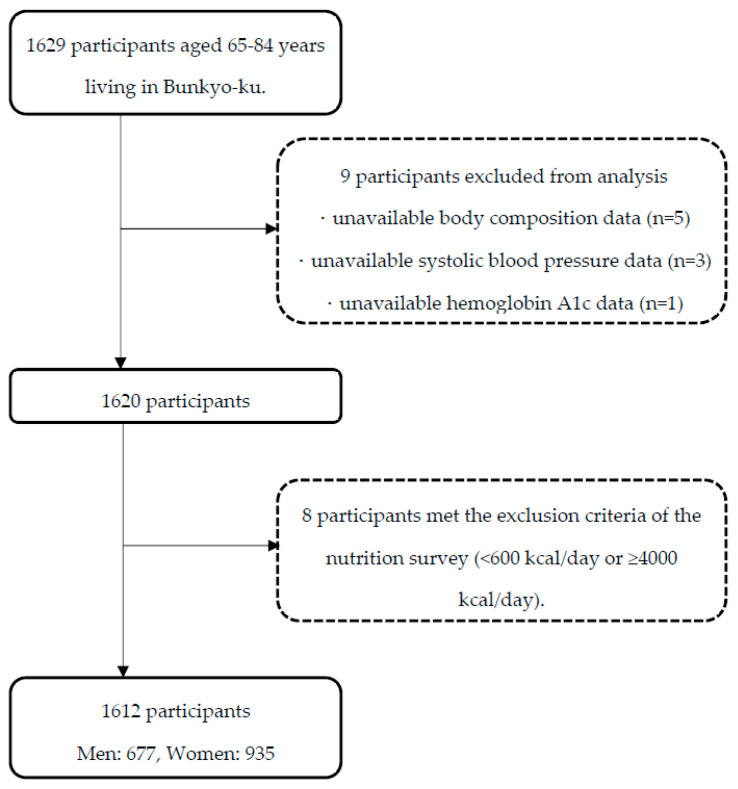
Flowchart of the participants.

As shown in Figure 1, we excluded nine of the 1629 participants enrolled in the Bunkyo Health Study, due to missing data (body composition [*n* = 5], systolic blood pressure [*n* = 3], hemoglobin A1c [*n* = 1]). Furthermore, of the remaining 1620 participants, eight who met the exclusion criteria of the nutrition survey [31] (<600 kcal/day or ≥4000 kcal/day) were excluded. Finally, 1612 participants (male: 677, female: 935) were included in this analysis.

The study protocol was approved by the ethics committee of Juntendo University in November 2015 (Nos. 2015078, 2016138, 2016131, 2017121, and 2019085). This research was conducted in accordance with the principles outlined in the Declaration of Helsinki. All participants provided written informed consent and were informed that they had the right to withdraw from the trial at any time.

### 2.2. Dietary Assessment

Dietary and nutrient intake were assessed using the brief-type self-administered diet history questionnaire (BDHQ) [32,33]. The BDHQ is a questionnaire printed on four A4-size pages and takes about 15 min to complete. The BDHQ asks about the frequency of dietary behaviors and consumption of 58 foods and beverages over the past month, including the frequency of daily consumption of 46 foods and nonalcoholic beverage items, rice, and miso soup, the frequency of consumption of five alcoholic beverages and the amount of each alcoholic beverage consumed per drinking occasion, and the frequency of daily consumption of five seasonings (salt, oil, sugar, soy sauce, and noodle soup) used in cooking and the general diet. Average daily food and nutrient intakes were estimated using an ad hoc computer algorithm for the BDHQ, based on the Standard Tables of Food Composition in Japan [34]. Food and nutrient intakes were energy-adjusted using the nutrient density method [35].

### 2.3. DPs

To identify DPs, we conducted a principal component analysis for each gender based on the energy-adjusted intake of 52 food and beverage items. In this analysis, we excluded the following six items as previously described: sugar added to coffee and black tea; salt, oil, and sugar used during cooking; table salt (and salt-containing seasonings); and soup consumed with noodles because they are considered cooking methods or seasonings/condiments [20,24,25]. We retained three factors for both men and women, considering eigenvalues, scree tests, and factor interpretability. The factor scores for each DP and for each individual were calculated by summing the intakes of the food items weighted by their factor loadings.

### 2.4. Genotyping

Genomic DNA was extracted from peripheral blood cells using a DNA extraction kit (DNeasy Blood and Tissue Kit; Qiagen, Fenlo, The Netherlands). We used the Illumina Infinium Asian Screening Array-24 v1.0 BeadChip (Illumina, San Diego, CA, USA) for *ALDH2* rs671 genotyping. Microarray scans were analyzed and genotyped with GenomeStudio (version 2013; Illumina, San Diego, CA, USA).

### 2.5. Other Measurements

Physical activity level was evaluated using the International Physical Activity Questionnaire (IPAQ) [36,37]. Brachial systolic and diastolic blood pressures were measured in the supine position after 10 min of rest. For biochemical tests, blood samples were collected in the morning after an overnight fast. All blood samples were tested at a contracted clinical laboratory (SRL, Tokyo, Japan). 

Hypertension was defined as systolic blood pressure ≥140 mmHg, diastolic blood pressure ≥90 mmHg, or the current use of antihypertensive medications. Diabetes mellitus was defined as hemoglobin A1c ≥ 6.5% plus either fasting blood glucose ≥126 mg/dL or 2 h blood glucose level ≥ 200 mg/dL after a 75 g oral glucose tolerance test or current use of diabetes medications. Dyslipidemia was defined as low-density lipoprotein (LDL) cholesterol ≥140 mg/dL, high-density lipoprotein (HDL) cholesterol <40 mg/dL, triglycerides ≥150 mg/dL, or current use of lipid-lowering agents.

### 2.6. Statistical Analyses

The Kolmogorov–Smirnov normality test was performed to evaluate the distribution of data. The participants were divided into two groups (*ALDH2* rs671 G/G carriers and *ALDH2* rs671 G/A or A/A carriers). Differences between genotypes and participant characteristics were compared by the unpaired t-test (for normally distributed variables), Mann–Whitney U-test (for non-normally distributed variables), and chi-squared test (for categorical variables). Normally and non-normally distributed variables are presented as means ± SD and medians (interquartile range), respectively, and categorical variables are presented as frequencies (percentages). Multiple regression analysis was performed to determine the independent contribution of the *ALDH2* rs671 G allele and alcohol intake (g/1000 kcal/day) to each DP. To adjust for potential covariates, we also added age, body mass index (BMI), years of education, smoking history, and physical activity as explanatory variables.

The Statistical Package for the Social Sciences v. 28.0 for Windows (SPSS, Chicago, IL, USA) was employed to analyze the data. All statistical tests were two-sided, with a 5% significance level.

## 3. Result

The median age of the subjects was 73 (68–77) years for both sexes, and the median BMI of men and women was 23.3 (21.7–25.1) and 22.0 (20.0–24.2), respectively. Three DPs were identified by principal component analysis in both men and women (Table 1). The first DP was named “Japanese side dish type” (DP1) because it was characterized by a high intake of fish, vegetables, potatoes, soy products, mushrooms, and fruits, and a low intake of rice. The second DP was characterized by a high intake of alcoholic beverages, seafood, and soy products, and a low intake of bread and confectioneries, and thus it was named “Japanese dish with alcohol type” (DP2). The third DP was the “Western dish with alcohol type” (DP3), characterized by a high intake of ham, pasta, mayonnaise, and alcoholic beverages, and a low intake of rice and miso soup. In men, these three main DPs accounted for 9.1%, 5.1%, and 4.1% of the variance in food intake, respectively, and together explained 18.3% of food intake variability. In women, they accounted for 8.4%, 4.9%, and 4.2% of the variance in food intake, respectively, and together explained 17.5% of food intake variability. 

As shown in Table 2, 371 (55%) male subjects had *ALDH2* rs671 G/G, 254 (37%) had G/A, and 52 (8%) had A/A. In women, the distribution was 520 (56%), 355 (38%), and 60 (6%), respectively. These genotype frequencies were similar to those previously reported for Japanese men and women []. Due to the small number of participants with *ALDH2* rs671 A/A, we divided these individuals into two groups: *ALDH2* rs671 G/G carriers and *ALDH2* rs671 G/A or A/A carriers. 

The anthropometric data of the participants with *ALDH2* rs671 G/G and *ALDH2* rs671 G/A or A/A are shown in Table 2. Men with *ALDH2* rs671 G/G had a significantly lower Brinkman index than men with *ALDH2* rs671 G/A or A/A. In addition, men with *ALDH2* rs671 G/G had significantly higher systolic blood pressure, diastolic blood pressure, fasting blood glucose, aspartate aminotransferase (AST), alanine aminotransferase (ALT), and γ-glutamyl transpeptidase (γ-GTP) than the men with *ALDH2* rs671 G/A or A/A. On the other hand, men with *ALDH2* rs671 G/G had significantly higher HDL cholesterol and lower LDL cholesterol than men with *ALDH2* rs671 G/A or A/A. Women with *ALDH2* rs671 G/G had significantly higher fasting blood glucose and γ-GTP than women with *ALDH2* rs671 G/A or A/A. By contrast, women with *ALDH2* rs671 G/G had significantly higher HDL cholesterol and lower LDL cholesterol.

The DP scores, nutrients, and food group intakes for each participant are shown in Table 3 according to the *ALDH2* genotype. Men with *ALDH2* rs671 G/G had significantly lower DP1 scores and significantly higher DP2 and DP3 scores than men with *ALDH2* rs671 G/A or A/A. Regarding nutrients, men with *ALDH2* rs671 G/G had significantly lower intakes of fat, carbohydrates, total dietary fiber, salt, and sugar (sucrose), and significantly higher intakes of alcohol and animal protein than men with *ALDH2* rs671 G/A or A/A. In terms of food groups, men with *ALDH2* rs671 G/G had significantly lower intakes of grain, sugar and sweeteners, green and yellow vegetables, fruits, and confectioneries, and significantly higher intake of beverages than men with *ALDH2* rs671 G/A or A/A. In women, DP2 and DP3 scores were significantly higher in individuals with *ALDH2* rs671 G/G than in those with *ALDH2* rs671 G/A or A/A. Regarding nutrients, women with *ALDH2* rs671 G/G had significantly lower intakes of fat, carbohydrates, and sucrose and significantly higher intakes of alcohol and animal protein than women with *ALDH2* rs671 G/A or A/A. In terms of food groups, only confectionery intake was significantly lower in women with *ALDH2* rs671 G/G compared to women with *ALDH2* rs671 G/A or A/A. These data suggest that in women, DP2 and DP3, and not DP1, were associated with the *ALDH2* rs671 genotype.

Next, to determine whether alcohol intake is an intermediate factor between the *ALDH2* genotype and DPs, we performed multiple regression analyses (Table 4 and Table 5). In men (Table 4), *ALDH2* rs671 was significantly correlated with the DP1 (β = 0.109, *p* = 0.004) and DP3 (β = −0.226, *p* < 0.001) scores in model 1. However, when we added alcohol intake as a covariate (model 2), alcohol intake was significantly correlated with these DP (DP1; β = −0.349, *p* < 0.001, DP3; β = 0.499, *p* < 0.001) scores, while *ALDH2* rs671 was not (DP1; β = −0.027, *p* = 0.483, DP3; β = −0.032, *p* = 0.361). *ALDH2* rs671 was also significantly correlated with the DP2 (β = −0.276, *p* < 0.001) score in model 1, and both *ALDH2* rs671 and alcohol intake were significantly correlated with the DP2 (*ALDH2* rs671; β = −0.092, *p* = 0.011, alcohol intake; β = 0.475, *p* < 0.001) score in model 2. In women (Table 5), *ALDH2* rs671 was not associated with the DP1 (β = 0.007, *p* = 0.818) score in model 1, while in model 2, alcohol intake was significantly correlated with the DP1 (β = −0.150, *p* < 0.001) score. *ALDH2* rs671 was significantly correlated with the DP2 (β = −0.236, *p* < 0.001) and DP3 (β = −0.107, *p* < 0.001) scores in model 1; however, these correlations were not observed in model 2 (DP2; β = −0.011, *p* = 0.630, DP3; β = 0.017, *p* = 0.588), and only alcohol intake was significantly correlated with these DP (DP2; β = 0.746, *p* < 0.001, DP3; β = 0.408, *p* < 0.001) scores. In addition, age and years of education were significantly correlated with all DP in men (age: DP1; β = 0.129, *p* < 0.001, DP3; β = −0.080, *p* = 0.015, years of education: DP1; β = 0.106, *p* = 0.003, DP2; β = −0.131, *p* < 0.001, DP3; β = 0.146, *p* < 0.001) and in women (age: DP1; β = 0.077, *p* = 0.026, DP2; β = 0.079, *p* < 0.001, DP3; β = −0.114, *p* < 0.001, years of education: DP1; β = 0.121, *p* < 0.001, DP2; β = −0.097, *p* < 0.001, DP3; β = 0.120, *p* < 0.001) scores in model 2 in both men and women, excluding the relationship between age and the DP2 score in men (β = 0.046, *p* = 0.174).

## 4. Discussion

We investigated the effects of *ALDH2* rs671 genotype and alcohol intake on DPs in elderly community-dwelling subjects. We identified three major DPs in both men and women based on principal component analysis: the “Japanese side dish type” (DP1), the “Japanese dish with alcohol type” (DP2), and the “Western dish with alcohol type” (DP3). In men, *ALDH2* rs671 was significantly associated with all DP scores. When alcohol intake was added as a covariate, *ALDH2* rs671 was still significantly correlated with the DP2 score, but not with the DP1 or DP3 score, and alcohol intake was significantly correlated with all DP scores. In women, *ALDH2* rs671 was significantly associated with the DP2 and DP3 scores; when alcohol intake was added as a covariate, however, those associations disappeared, and alcohol intake was significantly correlated with all DP scores.

The three major DPs identified in this study are consistent with those found in previous reports. The first DP defined in this study, the “Japanese side dish type” (DP1), is similar to the first DP described in many previous studies of Japanese people [5,20,22,23,25,38,39,40], which named it the “prudent type”, “healthy type”, or “side dish type”. In this study, DP2, the “Japanese dish with alcohol type”, and DP3, the “Western dish with alcohol type”, were characterized by alcohol intake. Concerningly, it has been shown that several DPs in young and middle-aged adults are characterized by alcohol intake. For example, previous reports identified DPs characterized by the intake of fish, seafood, and alcoholic beverages [20,25,26], or noodles and alcoholic beverages [21,22,41]; however, it remains unclear whether DPs are also characterized by alcohol intake in elderly people, who drink less than young people. This study is the first to show that alcohol intake is closely related to DPs even in elderly people.

While previous studies have demonstrated associations between the *ALDH2* genotype and several food preferences [14,15,18,19], this study is the first to reveal correlations between the *ALDH2* genotype and DPs. However, when adjusted for alcohol intake, most of these associations disappeared, and conversely, alcohol intake was significantly associated with each DP. Thus, the association between the *ALDH2* genotype and DPs seems to be strongly mediated by alcohol intake, and this is theoretically reasonable since the *ALDH2* genotype is strongly associated with alcohol consumption [10].

Several previous studies have shown that many DPs are characterized by alcohol consumption and that there is an inverse correlation between the intake of carbohydrates and alcohol [42]. In fact, people with higher alcohol intake were shown to have a lower intake of carbohydrates, protein, and fat [42,43,44]. In addition, the consumption of sweet foods was found to be increased during alcohol abstinence in people with alcohol dependence [45] or alcohol use disorders [46]. Two mechanisms have been suggested to explain the inverse relationship between carbohydrate intake and alcohol consumption. The first is that carbohydrates induce insulin secretion, which increases the activity of the serotonin system in the brain, thereby suppressing the preference for alcohol intake. The second is that the hedonic response to both alcohol and sweet consumption is mediated by the brain’s opioid system, and the consumption of one attenuates that of the other due to competition for receptors [47]. These results indicate that DPs may be more readily altered by alcohol intake than by *ALDH2* gene polymorphisms, which would have important clinical implications when considering alcohol-restricted dietary interventions. For example, DPs may change during such interventions, which could affect outcomes such as blood glucose levels and body weight [48].

On the other hand, this study showed that in men, the *ALDH2* genotype was significantly associated with DP2 independently of alcohol intake. In general, eating habits become more diverse as individuals enter their teens and 20 s, stabilize in their 30 s and 40 s, and are maintained thereafter [49]. Alcohol consumption is more strongly influenced by the *ALDH2* genotype in younger age groups [50]. Therefore, it is hypothesized that eating habits are formed between the 20 s and 40 s when alcohol intake is high, and then alcohol intake decreases with age; however, dietary habits in this study were not significantly affected by decreased alcohol intake. This may be why the *ALDH2* genotype was associated with DP2 independently of alcohol intake.

There are several limitations to the present study. First, the three DPs identified in this study comprised about 20% of DPs calculated for this population, and it is unclear whether *ALDH2* genotype and alcohol intake contribute to the other DPs. However, the explained variance ratio depends on the number of food items, and the smaller the number of food items analyzed, the larger the explained variance ratio [51]. Similar to this study, previous studies that identified DPs involving around 50 food items found that the cumulative explained variance ratio of three major DPs was around 20% [20,24,25]. In this study, DPs were calculated based on 52 food items; therefore, the cumulative explained variance ratio is considered reasonable and acceptable. Second, because of regional variations in the Japanese diet [52,53,54], it is unclear whether the analyzed DPs can be generalized to other regions. In particular, previous studies have suggested that elderly people in urban areas of Japan are more likely to consume alcohol than those in other areas of the country [55]. Third, the subjects in this study were elderly city-dwelling Japanese with a high educational background who may have had high health literacy [56]. Therefore, further studies are required to generalize these results.

## 5. Conclusions

In conclusion, the *ALDH2* genotype was associated with a variety of DPs in community-dwelling elderly people. However, most associations were mediated by alcohol intake as an intermediate factor.

## Figures and Tables

**Table 1:** Factor loading matrix for major dietary patterns identified by principal component analysis.

Food Groups	Men			Women		
	DP1	DP2	DP3	DP1	DP2	DP3
Law fat milk	0.193		−0.154	0.188		
Milk/yogurt					−0.155	
Chicken	0.183		0.179	0.194		
Pork/beef	0.197		0.264			
Ham/sausage/bacon		−0.279	0.271			0.338
Liver						
Squid/octopus/shrimps/shellfish		0.292			0.163	
Small fish with bone	0.279	0.261		0.290		−0.215
Canned tuna						
Dried/salted fish	0.192	0.417			0.204	
Oily fish	0.212	0.318		0.241	0.162	
Lean fish	0.220	0.288		0.262		
Egg	0.193		0.162	0.219		
Tofu/Deep-fried tofu	0.360	0.215		0.442	0.167	
Natto †	0.330	0.256		0.357	0.158	−0.162
Potatoes	0.396			0.326		
Pickled green leaves vegetable	0.277			0.279		
Other pickled vegetables	0.225	0.181			0.207	
Lettuces/cabbage (raw)	0.517	−0.189	0.303	0.533		0.293
Green leaves vegetable	0.594			0.589		
Cabbage/Chinese cabbage	0.548			0.594		
Carrots/pumpkin	0.650			0.629		
Japanese radish/turnip	0.553			0.492		
Other root vegetables	0.627			0.605		
Tomatoes	0.485	−0.173	0.225	0.437		0.187
Mushrooms	0.603			0.594		
Seaweeds	0.470	0.273	−0.215	0.516	0.182	
Western-type confectioneries		−0.477		−0.187	−0.438	
Japanese confectioneries		−0.312	−0.150	−0.161	−0.312	
Rice crackers/rice cake/okonomiyaki ‡		−0.335	−0.157	−0.227	−0.285	
Ice cream	−0.197	−0.225		−0.187	−0.223	0.202
Citrus fruit	0.353		−0.232	0.156	−0.179	
Persimmons/strawberries/kiwifruit	0.330		−0.225	0.243	−0.194	
Other fruit	0.361	−0.316	−0.195	0.294	−0.307	
Mayonnaise/dressing	0.190	−0.346	0.320			0.396
Bread		−0.550		−0.177	−0.387	0.262
Buckwheat noodles						
Japanese noodles					0.205	0.181
Chinese noodles	−0.221			−0.183		0.268
Pasta			0.168			0.302
Green tea	0.168		−0.300			−0.285
Black tea/oolong tea		−0.294			−0.172	0.163
Coffee		−0.223			−0.176	0.245
Cola drink/soft drink	−0.193	−0.168		−0.185		
100% fruit and vegetable juice						
Rice	−0.333		−0.642	−0.374	0.192	−0.652
Miso soup		0.225	−0.482		0.245	−0.480
Sake	−0.241	0.308	0.246		0.506	0.236
Beer	−0.227	0.375	0.374		0.485	0.299
Shochu	−0.238	0.369	0.324		0.539	0.276
Whisky	−0.218		0.234		0.406	0.245
Wine		0.172	0.349		0.437	0.301
Variance explained (%)	9.054	5.127	4.070	8.407	4.866	4.193

Factor loadings less than ± 0.15 are represented by a dash for simplicity. † Fermented soybeans. ‡ Savory pancake with various ingredients (meat, fish, and vegetable).

**Table 2:** Anthropometric characteristics of participants by *ALDH2* genotype.

	Men			Women		
	*ALDH2* rs671 (G/G)	*ALDH2* rs671 (G/A or A/A)	*p* Value	*ALDH2* rs671 (G/G)	*ALDH2* rs671 (G/A or A/A)	*p* Value
Number of Subjects	371 (55%)	306 G/A: 254 (37%), A/A: 52 (8%)		520 (56%)	415 G/A: 355 (38%), A/A: 60 (6%)	
Age (years)	72 (68–77)	73 (69–77)	0.503	73 (69–78)	72 (68–77)	0.111
BMI (kg/m^2^)	23.5 (21.9–25.2)	23.2 (21.6–25.1)	0.282	22.0 (20.1–24.1)	22.0 (19.9–24.2)	0.849
Physical activity (MET h/week)	5.5 (0.0–21.4)	8.9 (0.0–24.1)	0.093	5.3 (0.0–13.9)	4.8 (0.0–13.2)	0.426
Education (years)	16 (12–16)	16 (13–16)	0.123	12 (12–14)	12 (12–14)	0.332
Brinkman index	320.0 (0.0–760.0)	445.0 (0.8–888.8)	0.009	0.0 (0.0–0.0)	0.0 (0.0–0.0)	0.631
Smoking history (*n*/%)	263 (71%)	230 (75%)	0.213	92 (18%)	70 (17%)	0.741
Systolic BP (mmHg)	137.0 (126.0–149.0)	133.0 (124.0–144.3)	0.005	135.0 (123.0–148.0)	136.0 (126.0–148.0)	0.484
Diastolic BP (mmHg)	86.0 (80.0–93.0)	85.0 (80.0–91.0)	0.033	82.0 (76.0–89.0)	83.0 (76.0–90.0)	0.652
Fasting plasma glucose (mg/dL)	100.0 (93.0–111.0)	98.0 (92.0–107.0)	0.047	95.0 (90.0–103.0)	94.0 (88.0–100.0)	0.004
HbA1c (%)	5.7 (5.4–6.1)	5.8 (5.5–6.1)	0.141	5.7 (5.5–6.0)	5.7 (5.5–6.0)	0.652
Triglycerides (mg/dL)	88.0 (67.0–124.0)	93.5 (68.0–126.0)	0.692	80.0 (62.0–110.0)	85.0 (64.0–116.0)	0.064
HDL-C (mg/dL)	58.0 (49.0–68.0)	56.0 (47.0–66.0)	0.011	67.5 (58.0–79.0)	66.0 (57.0–77.0)	0.042
LDL-C (mg/dL)	109.0 (88.0–128.0)	117.0 (96.8–140.0)	<0.001	124.0 (104.0–143.8)	128.0 (108.0–150.0)	0.036
AST (IU/L)	22 (19–27)	21 (18–25)	0.003	22 (19–25)	22 (19–25)	0.549
ALT (IU/L)	18 (14–24)	17 (14–21)	0.019	16 (13–21)	16 (13–20)	0.609
γ-GTP (IU/L)	30 (20–47)	24 (18–37)	<0.001	19 (15–28)	18 (14–24)	0.005

BMI: body mass index, MET: metabolic equivalents, BP: blood pressure, HbA1c: hemoglobin A1c, HDL-C: high-density lipoprotein cholesterol, LDL-C: low-density lipoprotein cholesterol, AST: aspartate aminotransferase, ALT: alanine aminotransferase, γ-GTP: γ-glutamyl transferase. Data are expressed as means ± SD or medians (interquartile range). Data were analyzed using unpaired *t*-test (for distributed variables), Mann–Whitney *U* test (for non-normally distributed variables), or χ^2^ test (for categorical variables).

**Table 3:** Dietary pattern scores and nutrient and food group intake by *ALDH2* genotype in all participants.

	Men			Women		
	*ALDH2* rs671 (G/G)	*ALDH2* rs671 (G/A or A/A)	*p* Value	*ALDH2* rs671 (G/G)	*ALDH2* rs671 (G/A or A/A)	*p* Value
Number of Subjects	371 (55%)	306 G/A:254 (37%), A/A: 52 (8%)		520 (56%)	415 G/A: 355 (38%), A/A: 60 (6%)	
DP1 score	−0.16 (−0.74 to 0.48)	0.10 (−0.60 to 0.81)	<0.001	−0.13 (−0.69 to 0.55)	−0.04 (−0.69 to 0.56)	0.652
DP2 score	0.25 ± 0.93	−0.31 ± 1.00	<0.001	0.04 (−0.45 to 0.72)	−0.32 (−0.77 to 0.15)	<0.001
DP3 score	0.19 ± 0.99	−0.24 ± 0.96	<0.001	0.09 ± 0.99	−0.11 ± 1.00	0.004
Total energy intake (kcal)	2087 (1722–2538)	2012 (1635–2422)	0.176	1771 (1470–2144)	1798 (1469–2153)	0.686
Protein intake (% energy)	15.4 (13.5–17.2)	15.6 (13.7–17.9)	0.069	17.7 (15.5–19.8)	17.5 (15.6–19.9)	0.844
Fat intake (% energy)	25.8 (21.9–29.7)	27.8 (24.0–31.0)	<0.001	29.1 ± 5.4	29.9 ± 5.6	0.031
Carbohydrate intake (% energy)	46.3 (40.5–53.0)	52.3 (46.2–56.9)	<0.001	49.2 ± 7.8	51.3 ± 7.5	<0.001
Grain energy (% energy)	30.1 (24.2–38.1)	33.7 (26.3–41.5)	<0.001	28.3 (21.5–35.3)	28.3 (23.1–35.6)	0.148
Animal protein (% energy)	59.0 ± 9.3	57.5 ± 9.8	0.037	61.7 (55.2–67.1)	60.4 (53.4–66.2)	0.035
SFA (g/1000 kcal)	7.67 (6.29–8.83)	8.28 (6.96–9.73)	<0.001	8.79 ± 2.04	9.08 ± 2.05	0.032
MUFA (g/1000 kcal)	10.02 (8.53–11.82)	10.82 (9.28–12.40)	<0.001	11.35 ± 2.41	11.67 ± 2.55	0.052
Cholesterol (mg/1000 kcal)	204 (159–254)	211 (158–270)	0.268	249 (186–296)	244 (192–295)	0.870
n-6 PUFA (g/1000 kcal)	5.41 (4.59–6.46)	5.72 (4.86–6.68)	0.025	5.94 ± 1.33	6.13 ± 1.37	0.034
n-3 PUFA (g/1000 kcal)	1.53 (1.21–1.81)	1.50 (1.22–1.84)	0.923	1.66 (1.41–2.05)	1.68 (1.4–2.05)	0.991
Total dietary fiber (g/1000 kcal)	6.5 (5.3–7.8)	7.2 (5.9–8.7)	<0.001	8.4 (6.9–9.9)	8.4 (7.2–10.0)	0.184
Salt (g/1000 kcal)	6.1 (5.4–6.8)	6.3 (5.5–7.1)	0.011	6.5 (5.7–7.5)	6.5 (5.6–7.4)	0.579
Sugar (sucrose) (g/1000 kcal)	5.2 (3.2–8.0)	7.4 (4.4–10.5)	<0.001	6.8 (4.3–10.0)	8.1 (4.7–11.3)	0.002
Alcohol (g/1000 kcal)	12.2 (4.4–22.9)	0.5 (0.0–7.2)	<0.001	0.6 (0.0–6.2)	0.0 (0.0–0.1)	<0.001
Alcohol (g)	24.7 (8.8–49.6)	0.9 (0.0–14.7)	<0.001	1.1 (0.0–10.4)	0.0 (0.0–0.2)	<0.001
Grains (g/1000 kcal)	171.5 (134.1–220.5)	187.4 (147.0–233.2)	0.003	155.5 (114.9–198.2)	159.6 (125.5–198.0)	0.266
Potatoes (g/1000 kcal)	15.1 (8.2–30.3)	17.0 (7.6–33.4)	0.325	25.1 (12.0–41.2)	25.8 (12.2–44.4)	0.634
Sugars and sweeteners (g/1000 kcal)	2.1 (1.2–3.3)	2.5 (1.5–4.3)	<0.001	2.8 (1.7–4.2)	2.7 (1.7–4.2)	0.539
Beans (g/1000 kcal)	31.0 (18.8–50.2)	30.4 (16.9–48.9)	0.412	39.3 (24.3–58.1)	40.0 (23.3–60.1)	0.970
Green and yellow vegetables (g/1000 kcal)	55.4 (32.6–82.7)	65.0 (41.7–94.8)	0.004	77.6 (55.3–112.8)	79.6 (57.1–109.2)	0.471
Other vegetables (g/1000 kcal)	80.7 (57.1–112.4)	90.5 (62.4–122.6)	0.076	119.1 (88.9–154.5)	119.0 (88.5–156.9)	0.958
Fruits (g/1000 kcal)	55.0 (32.4–89.9)	73.7 (39.3–119.3)	<0.001	85.1 (51.4–123.4)	92.4 (55.5–135.2)	0.050
Fish and shellfish (g/1000 kcal)	44.5 (33.0–63.5)	41.5 (27.8–62.0)	0.086	52.5 (36.4–75.0)	50.7 (34.6–72.7)	0.423
Meats (g/1000 kcal)	34.1 (25.7–45.9)	36.8 (25.8–48.0)	0.347	41.3 (28.7–54.7)	39.4 (28.4–54.2)	0.390
Eggs (g/1000 kcal)	17.9 (10.5–30.5)	18.6 (9.7–32.2)	0.824	23.7 (12.6–33.9)	22.6 (12.8–33.4)	0.993
Dairy products (g/1000 kcal)	81.7 (45.3–111.0)	86.2 (45.0–123.6)	0.183	93.2 (58.7–129.9)	92.8 (64.5–131.6)	0.850
Fats and oils (g/1000 kcal)	5.5 (3.9–7.0)	5.3 (4.1–7.0)	0.822	5.2 (3.7–6.7)	5.4 (3.8–7.2)	0.079
Confectioneries (g/1000 kcal)	14.9 (7.2–29.0)	23.0 (12.3–35.6)	<0.001	24.4 (12.0–37.5)	27.0 (14.4–44.3)	0.021
Beverages (g/1000 kcal)	420.7(316.5–553.9)	371.0 (266.8–506.1)	<0.001	389.4 (276.3–513.4)	375.8 (262.4–511.6)	0.384
Seasonings (g/1000 kcal)	125.3 (90.0–169.6)	127.4 (96.6–168.7)	0.228	106.5 (75.2–152.4)	106.8 (79.2–148.7)	0.733

DP: dietary pattern, SFA: saturated fatty acid, MUFA: monounsaturated fatty acid, PUFA: polyunsaturated fatty acid. Data are expressed as means ± SD or medians (interquartile range). Data were analyzed using unpaired *t*-test (for distributed variables) or Mann–Whitney *U* test (for non-normally distributed variables).

**Table 4:** Effect of *ALDH2* genotype and alcohol consumption on dietary pattern scores in men.

Dependent Variable	Independent Variable	B	Std. Error	β	*p*
DP1	Age (years)	0.031	0.007	0.163	<0.001
Model 1	BMI (kg/m^2^)	0.013	0.014	0.035	0.354
(R^2^ = 0.067)	Physical activity (MET h/week)	0.009	0.002	0.163	<0.001
	Education (years)	0.039	0.015	0.098	0.011
	Smoking history (*n*/%)	−0.132	0.084	−0.059	0.116
	*ALDH2* rs671 (G/G or G/A and A/A)	0.219	0.075	0.109	0.004
DP1	Age (years)	0.024	0.007	0.129	<0.001
Model 2	BMI (kg/m^2^)	0.007	0.013	0.019	0.600
(R^2^ = 0.167)	Physical activity (MET h/week)	0.009	0.002	0.156	<0.001
	Education (years)	0.043	0.015	0.106	0.003
	Smoking history (*n*/%)	−0.020	0.080	−0.009	0.807
	*ALDH2* rs671 (G/G or G/A and A/A)	−0.054	0.077	−0.027	0.483
	Alcohol (g/day)	−0.027	0.003	−0.349	<0.001
DP2	Age (years)	0.000	0.007	0.000	0.990
Model 1	BMI (kg/m^2^)	0.004	0.013	0.012	0.754
(R^2^ = 0.089)	Physical activity (MET h/week)	0.002	0.002	0.038	0.309
	Education (years)	−0.048	0.015	−0.120	0.002
	Smoking history (*n*/%)	0.135	0.083	0.060	0.103
	*ALDH2* rs671 (G/G or G/A and A/A)	−0.555	0.074	−0.276	<0.001
DP2	Age (years)	0.009	0.006	0.046	0.174
Model 2	BMI (kg/m^2^)	0.012	0.012	0.034	0.310
(R^2^ = 0.275)	Physical activity (MET h/week)	0.003	0.002	0.047	0.151
	Education (years)	−0.053	0.014	−0.131	<0.001
	Smoking history (*n*/%)	−0.018	0.075	−0.008	0.814
	*ALDH2* rs671 (G/G or G/A and A/A)	−0.184	0.072	−0.092	0.011
	Alcohol (g/day)	0.037	0.003	0.475	<0.001
DP3	Age (years)	−0.024	0.007	−0.129	<0.001
Model 1	BMI (kg/m^2^)	−0.007	0.013	−0.019	0.610
(R^2^ = 0.091)	Physical activity (MET h/week)	0.002	0.002	0.031	0.400
	Education (years)	0.063	0.015	0.157	<0.001
	Smoking history (*n*/%)	0.102	0.083	0.045	0.220
	*ALDH2* rs671 (G/G or G/A and A/A)	−0.454	0.074	−0.226	<0.001
DP3	Age (years)	−0.015	0.006	−0.080	0.015
Model 2	BMI (kg/m^2^)	0.002	0.012	0.004	0.897
(R^2^ = 0.296)	Physical activity (MET h/week)	0.002	0.002	0.041	0.203
	Education (years)	0.059	0.013	0.146	<0.001
	Smoking history (*n*/%)	−0.059	0.074	−0.026	0.425
	*ALDH2* rs671 (G/G or G/A and A/A)	−0.065	0.071	−0.032	0.361
	Alcohol (g/day)	0.039	0.003	0.499	<0.001

DP: dietary pattern, BMI: body mass index, MET: metabolic equivalents. B: partial regression coefficients B, Std. Error: standard error of partial regression coefficients B, β: standardized partial regression coefficients β.

**Table 5:** Effect of *ALDH2* genotype and alcohol consumption on dietary pattern scores in women.

Dependent Variable	Independent Variable	B	Std. Error	β	*p*
DP1	Age (years)	0.016	0.006	0.087	0.012
Model 1	BMI (kg/m^2^)	−0.012	0.010	−0.037	0.264
(R^2^ = 0.024)	Physical activity (MET h/week)	0.005	0.003	0.059	0.073
	Education (years)	0.057	0.016	0.124	<0.001
	Smoking history (*n*/%)	−0.210	0.086	−0.080	0.015
	*ALDH2* rs671 (G/G or G/A and A/A)	0.015	0.065	0.007	0.818
DP1	Age (years)	0.014	0.006	0.077	0.026
Model 2	BMI (kg/m^2^)	−0.011	0.010	−0.035	0.277
(R^2^ = 0.042)	Physical activity (MET h/week)	0.005	0.003	0.064	0.046
	Education (years)	0.055	0.016	0.121	<0.001
	Smoking history (*n*/%)	−0.132	0.087	−0.050	0.131
	*ALDH2* rs671 (G/G or G/A and A/A)	−0.076	0.068	−0.038	0.264
	Alcohol (g/day)	−0.022	0.005	−0.150	<0.001
DP2	Age (years)	0.005	0.006	0.028	0.403
Model 1	BMI (kg/m^2^)	0.013	0.010	0.042	0.185
(R^2^ = 0.093)	Physical activity (MET h/week)	−0.001	0.002	−0.018	0.576
	Education (years)	−0.052	0.015	−0.113	<0.001
	Smoking history (*n*/%)	0.384	0.083	0.145	<0.001
	*ALDH2* rs671 (G/G or G/A and A/A)	−0.475	0.063	−0.236	<0.001
DP2	Age (years)	0.014	0.004	0.079	<0.001
Model 2	BMI (kg/m^2^)	0.011	0.007	0.035	0.102
(R^2^ = 0.575)	Physical activity (MET h/week)	−0.004	0.002	−0.047	0.028
	Education (years)	−0.044	0.010	−0.097	<0.001
	Smoking history (*n*/%)	−0.007	0.058	−0.003	0.907
	*ALDH2* rs671 (G/G or G/A and A/A)	−0.022	0.045	−0.011	0.630
	Alcohol (g/day)	0.111	0.003	0.746	<0.001
DP3	Age (years)	−0.026	0.006	−0.142	<0.001
Model 1	BMI (kg/m^2^)	0.012	0.010	0.037	0.251
(R^2^ = 0.068)	Physical activity (MET h/week)	0.004	0.003	0.052	0.102
	Education (years)	0.051	0.015	0.111	<0.001
	Smoking history (*n*/%)	0.333	0.084	0.126	<0.001
	*ALDH2* rs671 (G/G or G/A and A/A)	−0.215	0.064	−0.107	<0.001
DP3	Age (years)	−0.021	0.006	−0.114	<0.001
Model 2	BMI (kg/m^2^)	0.010	0.009	0.033	0.260
(R^2^ = 0.212)	Physical activity (MET h/week)	0.003	0.002	0.036	0.222
	Education (years)	0.055	0.014	0.120	<0.001
	Smoking history (*n*/%)	0.119	0.079	0.045	0.133
	*ALDH2* rs671 (G/G or G/A and A/A)	0.033	0.062	0.017	0.588
	Alcohol (g/day)	0.061	0.005	0.408	<0.001

DP: dietary pattern, BMI: body mass index, MET: metabolic equivalents. B: partial regression coefficients B, Std. Error: standard error of partial regression coefficients B, β: standardized partial regression coefficients β.

## Data Availability

Some or all datasets generated and/or analyzed during the current study are not publicly available; however, they can be obtained from the corresponding author upon a reasonable request.
